# Induction of annexin-1 at transcriptional and post-transcriptional level in rat brain by methylprednisolone and the 21-aminosteroid U74389F

**DOI:** 10.1155/S0962935196000531

**Published:** 1996-10

**Authors:** P. H. Voermans, K. G. Go, G. J. Ter Horst, M. H. J. Ruiters, E. Solito, L. Parente

**Affiliations:** 1 Department of Neurosurgery University Hospital Groningen Hanzeplein. 1 P.O. Box 30.001 Groningen NL-9700 RB The Netherlands; 2 Department of Biological Psychiatry University Hospital Groningen Hanzeplein. 1 P.O. Box 30.001 Groningen NL-9700 RB The Netherlands; 3 BioMedical Technology Center University Hospital Groningen Hanzeplein. 1 P.O. Box 30.001 Groningen NL-9700 RB The Netherlands; 4 Institut Cochin de Génétique Moléculaire Paris France; 5 Istituto di Farmacología e Farmacognosía Palermo Italy

## Abstract

Brain tissue of rats pretreated with methylprednisolone or with the 21-aminosteroid U74389F, and that of untreated control rats, was assessed for the expression of annexin-1 (Anx-1) and the transcription of its mRNA. For this purpose Anx-1 cDNA was amplified and simultaneously a T7-RNA-polymerase promoter was incorporated into the cDNA using a polymerase chain reaction (PCR). Then digoxigenin-11-UTP was incorporated into the transcribed cRNA with T7-RNA-polymerase. With this probe *in situ* hybridization was carried out on sections of the brain. The probe was visualized by an immunoassay using an antidigoxigenin antibody conjugate. Anx-1 protein was assessed by means of immunohistochemistry using a polyclonal antibody. The various brain areas of the control animals showed an appreciable amount of Anx-1 at mRNA or protein level; on the other hand, the animals which had been pretreated with either steroid, showed a more intense Anx-1 mRNA signal than the controls in many areas. In the pretreated animals Anx-1 immunostaining was unchanged in cortex, basal ganglia, amygdala and septum, but more intense in hippocampus, hypothalamus and thalamus. In ependyma, choroid plexus, meninges, and vascular walls there was no Anx-1 mRNA transcription detectable. An opposite profile was shown by the Anx-1 immunoreactivity, the protein was present in control animals as well as the steroid-pretreated animals, suggesting that here the protein was either from systemic origin, or has diffused from adjacent structures. The results indicated that Anx-1 mRNA transcription is upregulated by either steroid, and that in the untreated animals there is a resting level of Anx-1 mRNA transcription, presumably reflecting physiological influences on Anx-1 expression.


Mediators of Inflammation, 5, 370–378 (1996)

### Induction of annexin-1 at transcriptional and post-transcriptional level in rat brain by methylprednisolone and the 21-aminosteroid U74389F

P. H. Voermans,[1] K. G. Go,[1,CA] G. J. Ter Horst,[2] M. H. J. Ruiters,[3] E. Solito[4] and L. Parente[5]

Departments of [1]Neurosurgery, [2]Biological Psychiatry, and [3]BioMedical Technology Center, University Hospital Groningen, Hanzeplein 1, P.O. Box 30.001, NL-9700 RB Groningen, The Netherlands; [4]Institut Cochin de Génétique Moléculaire, Paris, France; and [5]Istituto di Farmacología e Farmacognosía, Palermo, Italy

[CA]Corresponding Author
Tel: (+31) 50 3612442
Fax: (+31) 50 3611715

**Brain tissue of rats pretreated with methylprednisolone or with the 21-aminosteroid U74389F, and that of untreated control rats, was assessed for the expression of annexin-1 (Anx-1) and the transcription of its mRNA. For this purpose Anx-1 cDNA was amplified and simultaneously a T7-RNA-polymerase promoter was incorporated into the cDNA using a polymerase chain reaction (PCR). Then digoxigenin-11-UTP was incorporated into the transcribed cRNA with T7-RNA-polymerase. With this probe *in situ* hybridization was carried out on sections of the brain. The probe was visualized by an immunoassay using an anti-digoxigenin antibody conjugate. Anx-1 protein was assessed by means of immunohistochemistry using a polyclonal antibody. The various brain areas of the control animals showed an appreciable amount of Anx-1 at mRNA or protein level; on the other hand, the animals which had been pretreated with either steroid, showed a more intense Anx-1 mRNA signal than the controls in many areas. In the pretreated animals Anx-1 immunostaining was unchanged in cortex, basal ganglia, amygdala and septum, but more intense in hippocampus, hypothalamus and thalamus. In ependyma, choroid plexus, meninges, and vascular walls there was no Anx-1 mRNA transcription detectable. An opposite profile was shown by the Anx-1 immunoreactivity, the protein was present in control animals as well as the steroid-pretreated animals, suggesting that here the protein was either from systemic origin, or has diffused from adjacent structures. The results indicated that Anx-1 mRNA transcription is upregulated by either steroid, and that in the untreated animals there is a resting level of Anx-1 mRNA transcription, presumably reflecting physiological influences on Anx-1 expression.**

**Key words:** Annexin-1, *In situ* hybridization, Lipocortin, PCR, Steroids

### Introduction

Since the 1960s glucocorticoidsteroids have been successfully employed in the clinical treatment of vasogenic brain oedema. Oedema of this type originates from disruption of the blood–brain barrier, resulting in exudation of blood plasma into the brain parenchyma. The vasogenic oedema and the inherent disruption of the blood–brain barrier tends to accompany most focal lesions of the brain, such as tumours, inflammatory lesions, infarctions, trauma and haemorrhage. The disruption of the barrier which is to be localized in the endothelium of the cerebral capillaries, has been ascribed to changes of endothelial permeability, allegedly caused by oedema mediators, although the barrier defect in brain tumours is usually due to the occurrence of fenestrations in the endothelial cytoplasm.[1] Oedema mediators include free unsaturated fatty acids, especially arachidonic acid, and oxygen derived free radicals, which have been shown to be produced in brain tissue during injury. The unsaturated fatty acids are released from membrane phospholipids by phospholipase $A_2$ attacking the glycerophospholipid molecule at the sn-2 position. Moreover, arachidonic acid, and its derivatives the eicosanoids, are allegedly involved in inflammatory processes in other tissues of the body.

Annexins, or lipocortins, have been detected as mediators of the anti-inflammatory action of glucocorticosteroids by inhibiting phospholipase $A_2$.[2,3] This inhibition is both calcium- and

© 1996 Rapid Science Publishers lipid-dependent.[4–6] Upon intravenous injection, Anx-1, 2 and 5 proved to exert a potent anti-inflammatory effect in rat and mouse.[7,8] The annexins are a family of proteins which all possess a conserved 70 amino acid repeating unit. In most annexins, there is a four-fold repeat, except for Anx-6 which has eight such repeats. Moreover, all annexins possess $Ca^{2+}$ and phospholipid binding sites. The differences between the annexin proteins are mainly located in the amino-terminus.[9]

Anx-1 has been shown to be abundantly present in extracts of human placenta, and in the spleen, and kidneys of the rat. Readily detectable tissue levels in the rat are found in the lung, the thymus, the liver, the heart, the brain, the bone marrow and the ileum. In a previous immunocytochemical study on rat brain, annexin-immunoreactivity only appeared in sporadic microglial cells and in the choroid plexus, being absent in the greater part of the brain in animals not pretreated with steroids. However, after administration of methylprednisolone or the 21-aminosteroid U74389F the neurones, ependyma, oligodendroglia and capillary endothelium of the brain showed an induction of annexin expression.[10]

It has been the aim of the present investigation to assess in the central nervous system the expression of Anx-1 by methylprednisolone and the 21-aminosteroid U74389F, mediated by the transcription of Anx-1 mRNA, thereby excluding the presence of Anx-1 which has originated from peripheral sources.

### Materials and Methods

### Oligonucleotide primers and promoters

The sequences of the oligonucleotides used in this study and the predicted lengths of the PCR amplification products are listed in Table 1. All oligonucleotides were prepared at the BioMedical Technology Centre of the Academic Hospital Groningen.

### Polymerase chain reaction (PCR)

Circa 10 ng annexin-1 (Anx-1) human cDNA was used as PCR template. The use of human cDNA is justified by the 87% homology with the murine cDNA.[11] The PCR mixture consisted of 10 μl PCR buffer (500 mM KCl, 15 mM $MgCl_2$ and 100 mM Tris-HCl pH 9.0), 1 nmol start and stop primers, 10 μl dNTP mixture (2 mM dATP, dTTP, dGTP, dCTP) and sterile $H_2O$ to a final volume of 100 μl. After preheating the PCR mixture for 5 min at 94°C, 2.5 U Taq DNA polymerase (Pharmacia Biotech, Uppsala, Sweden) was added. Then the mixture was covered with 100 μl sterile paraffin oil. The PCR profile was 94°C for 1 min, 60°C for 1 min and 72°C for 1 min for 30 cycles in a thermal cycler (Pharmacia LKB Gene ATAQ Controller). Amplified DNA was analysed by agarose gel electrophoresis and ethidium bromide staining using standard techniques.

### Transcription of digoxigenin-conjugated cRNA

All reactions involving the use of RNA were performed with solutions treated with 0.1% diethylpyrocarbonate (DEPC). Labelled cRNA was produced with a T7-RNA-polymerase (Pharmacia Biotech, Uppsala, Sweden) reaction to which digoxigenin-11-UTP (Boehringer Mannheim, Mannheim, Germany) was added. The following reaction mixture was incubated at 37°C for 2 h: 1 μg PCR-product, 2 μl transcription buffer (400 mM Tris-HCl pH 8.0, 60 mM $MgCl_2$, 20 mM spermidine and 100 mM NaCl), 2 μl NTP-mixture (10 mM ATP, CTP, GTP, 6.5 mM UTP and 3.5 mM digoxigenin-11-UTP), 63 U T7-RNA-polymerase and DEPC treated $H_2O$ to a final volume of 20 μl. The reaction was arrested by the addition of 2 μl of 0.2 M EDTA pH 8.0, followed by RNA extraction and pre-

**Table 1.** Sequences of polymerase chain reaction primers (promoter sequence after Young *et al.* (1993)[12]

| Primer | Sequence | Predicted PCR product length (base pairs) |
|---|---|---|
| Antisense | | |
| PV5 | 5′-ATGGCAATGGTATCAGAATTCCTC-3′ | |
| T7-PV3 | 5′-ATTAATACGACTCACTATAGGG-PV3-3′ | 1088 |
| Sense | | |
| PV3 | 5′-TTAGTTTCCTCCACAAAGAGCCAC-3′ | |
| T7-PV5 | 5′-ATTAATACGACTCACTATAGGGT-PV5-3′ | 1089 |

cipitation. The transcripts were subjected to agarose gel electrophoresis and Northern blotting and identified by immunoassay using an anti-digoxigenin antibody conjugate (Boehringer Mannheim, Mannheim, Germany) following the manufacturer's instructions.

### Steroids and dosage

Adult Wistar rats with a mean body weight of 350–400 g, which had free access to food and water were used in the studies. They were divided into three experimental groups: untreated control animals ($n = 7$), a group treated with 2 mg/kg methylprednisolone (Solumedrol, UpJohn Co., Kalamazoo, USA) dissolved in benzyl alcohol ($n = 8$), and a group treated with the 21-aminosteroid U74389F (UpJohn Co., Kalamazoo, USA) dissolved in saline solution ($n = 4$). All drugs were administered 24 and 2 h before the animals were killed. The animals were anaesthetized with ether and perfused with 0.9% NaCl and 4% paraformaldehyde in 0.1 M phosphate buffered saline (PBS) (pH 7.4). The brains were removed and cryoprotected overnight in a 10% sucrose solution. The brains were quickly frozen in $CO_2$ and sections of 40 μm thickness were cut on a cryostat microtome. The sections were stored in antifreeze (50% PBS, 30% ethylene glycol, 20% glycerol) at −20°C. The sections were mounted on poly-L-lysine coated slides, air dried overnight and then used for *in situ* hybridization (ISH).

### *In situ* hybridization

The slides were incubated in a Proteinase K solution (10 μg/ml in 10 mM Tris, 1 mM EDTA pH 7.5) for 10 min at 37°C, rinsed twice in PBS and dehydrated in ethanol. On the slides 100 μl of hybridization mixture was applied. The hybridization mixture consisted of 4*SSC (1*SSC is 0.15 M NaCl and 0.015 M sodium citrate), 1*Denhardt's (0.02% Ficoll, 0.02% polyvinylpyrodilone, 10 mg/ml BSA (Section V)), 50% deionized formamide, 5% dextran sulphate, 0.5 mg/ml tRNA and 1 μg/ml digoxigenin-labelled cRNA probe. The slides were covered with coverslips and then sealed in a humid plastic bag. Hybridization was conducted overnight at 65°C. Coverslips were removed with 4*SSC rinsing. Posthybridization washes were as follows: 2*SSC, 1*SSC, 0.5*SSC and 0.1*SSC for 30 min at room temperature. To compare the results in the various groups the ISH procedure was carried out simultaneously for all the groups.

### Immunological detection of digoxigenin-labelled cRNA

The slides were rinsed in a solution containing 100 mM Tris and 150 mM NaCl (pH 7.4) (buffer 1) for 1 min at room temperature and incubated for 30 min in 1% blocking agent in buffer 1. They were then washed for 1 min in buffer 1 and subsequently incubated with anti-digoxigenin alkaline phosphatase conjugated antibody (1:500 diluted in buffer 1). The slides were washed twice in buffer 1 for 15 min at room temperature and equilibrated for 2 min in 100 mM Tris, 100 mM NaCl, 50 mM $MgCl_2$ (pH 9.5) (buffer 2). The presence of the phosphatase on the slides was revealed with a fresh colour substrate solution, containing 45 μl 90 mM nitro blue tetrazolium salt (NBT) (Sigma, Bornem, Belgium) in 70% (v/v) dimethylformamide, and 35 μl, 120 mM 5-bromo-4-chloro-3-indolylphosphate toludinium salt in 100% dimethylformamide (BCIP) (Sigma, Bornem, Belgium) in 10 ml of buffer 2. The reaction was stopped in 10 mM Tris, 1 mM EDTA (pH 7.5). The sections were embedded with Aquamount (BDH, Dorset, UK).

### Immunocytochemical labelling of Anx-1

Free-floating 40 μm thick sections of the brain were washed three times with PBS and subsequently incubated overnight with a polyclonal rabbit antibody raised against the amino terminus of Anx-1.[1] The antibody was diluted 1:40 in PBS and the solution also contained 2% BSA and 10% normal goat serum. After three washes with PBS the sections were incubated with the secondary antibody (goat anti-rabbit 1:800) (Sigma, Bornem, Belgium). After secondary incubation, the sections were rinsed in PBS and incubated in rabbit peroxidase anti-peroxidase 1:500 (Dakopatts, Denmark). Following the last incubation, the sections were rinsed in PBS and 0.1 M sodium-acetate (pH 6.0). The reaction was developed in 0.1 M sodium-acetate (pH 6.0) containing 0.05% of the chromagen 3,3′-diaminobenzidine, 2.5% ammonium nickel sulphate, 0.4% ammonium chloride and 0.01% $H_2O_2$. Staining was arrested by rinsing with 0.1 M sodium-acetate (pH 6.0). After staining, the sections were mounted on gelatin-chrom-alum-coated slides, air-dried, dehydrated in a graded ethanol series and xylol, and coverslipped with DePeX (BDH, Dorset, UK). To allow comparison of the various groups of animals, the immunocytochemistry was carried out in the same session to ensure similar reaction times. Moreover, the tissue sections employed for immunohistochemical detection of Anx-1 were adjacent to those used for ISH of Anx-1 mRNA.

### Analysis of results

The sections used in the ISH and immunocytochemistry studies were analysed by two observers, who were unaware of the treatment of the rats. The relative intensities of the hybridization signal in the different brain regions were judged as weak (+), strong (++), or very strong (+++) (Tables 2 and 3). No attempt was made to quantify the differences between the treated animals and controls.

### Results

### Production of labelled cRNAs

By the inclusion of the T7-RNA-polymerase promoter sequence in the 5′-termini of the PCR primers (Table 1) the promoter was incorporated into the terminus of the Anx-1 cDNA amplification products. Separate cDNA templates from which antisense and sense cDNAs could be transcribed, were generated for the Annexin-1 gene; and DNA fragments of the predicted size could be demonstrated by restriction and subsequent electrophoretic analysis of the amplification products. Moreover, Northern blot analysis showed discrete digoxigenin-conjugated sense and antisense cRNAs, which were transcribed from the amplification products by the T7-RNA-polymerase. The identities of the cRNAs were confirmed using the template cDNA and unlabelled cRNA as positive controls to which the labelled Anx-1 cRNA specifically hybridized.

### *In situ* hybridization (ISH)

Using the digoxigenin-labelled antisense cRNA probe for Anx-1 mRNA, *in situ* hybridization (ISH) on paraformaldehyde-fixed frozen sections of rat brains resulted in an adequate colour reaction with minimal background staining. Both the corresponding sense probe hybridization experiment and the ISH, which had been performed with the digoxigenin-labelled antisense neo-RNA were negative, confirming the specificity of the Anx-1 cRNA hybridization reaction.

**Table 2.** Comparison between the subjective evaluations of Anx-1 mRNA ISH signal distribution in control animals and animals treated with methylprednisolone or the 21-aminosteroid, and occurrence of glucocorticoid receptor (GR) immunoreactivity

| Structure | Control | Methyl-prednisolone | 21-amino-steroid | GR-immuno reactivity[a] |
|---|---|---|---|---|
| *Telencephalon* | | | | |
| Caudate-putamen | + | ++ | ++ | +++ |
| Cortex | | | | |
| Layer 1 | 0 | 0 | 0 | 0/+ |
| Layer 2–6 | + | ++ | ++ | +++ |
| Olfactory bulb | + | ++ | ++ | ++ |
| Amygdala | + | ++ | ++ | ++ |
| Corpus callosum | | | | |
| fibres | 0 | 0 | 0 | |
| oligodendroglia | + | + | + | |
| Hippocampus | | | | |
| CA1 | + | +++ | +++ | +++ |
| CA2 | + | +++ | +++ | +++ |
| CA3 | + | +++ | ++ | ++ |
| Dentate gyrus granular layer | + | +++ | +++ | +++ |
| Fimbria hippocampi | 0 | 0 | 0 | |
| Septum | + | ++ | ++ | ++ |
| Bed nucleus striae terminalis | + | ++ | ++ | ++ |
| *Diencephalon* | | | | |
| Hypothalamus | + | ++ | ++ | ++ |
| Thalamus | + | ++ | ++ | ++ |
| Epithalamus | | | | |
| habenular nucleus | + | +++ | +++ | + |
| *Non-parenchymal tissue* | | | | |
| Vascular wall | 0 | 0 | 0 | |
| Meninges | 0 | 0 | 0 | |
| Choroid plexus | 0 | 0 | 0 | |
| Ventricular ependymocytes | 0 | 0 | 0 | |

The scores of subjective evaluations were ranked as follows: 0 = no signal/cells labelled, + = weak signal, ++ = strong signal, +++ = very strong signal.
[a]Reference: Cintra *et al.* (1994).[24]

**Table 3.** Comparison of the immunoreactive Anx-1 distribution in control animals and animals treated with methylprednisolone or the 21-aminosteroid, and the occurrence of immunoreactive annexin-1

| Structure | Control | Methyl-prednisolone | 21-amino-steroid | Literature[a] |
|---|---|---|---|---|
| *Telencephalon* | | | | |
| Caudate-putamen | ++ | ++ | ++ | nd |
| Cortex | | | | ++ |
| layer 1 | + | + | + | |
| layer 2 | ++ | ++ | ++ | |
| layer 3, 4 | + | + | + | |
| layer 5 | ++ | ++ | ++ | |
| layer 6 | + | + | + | |
| Olfactory bulb | + | + | + | nd |
| Amygdala | + | + | + | ++ |
| Corpus callosum | | | | nd |
| Fibres | + | + | + | |
| Oligodendroglia | 0 | 0 | 0 | |
| Hippocampus | | | | |
| CA1 | + | ++ | ++ | ++ |
| CA2 | + | ++ | ++ | ++ |
| CA3 | + | +++ | ++ | ++ |
| Dentate gyrus granular layer | + | ++ | ++ | ++ |
| Fimbria hippocampi | 0 | 0 | 0 | nd |
| Septum | + | + | + | ++ |
| Bed nucleus striae terminalis | + | + | + | nd |
| *Diencephalon* | | | | |
| Hypothalamus | + | ++ | ++ | ++ |
| Thalamus | + | ++ | ++ | ++ |
| Epithalamus | | | | nd |
| habenular nucleus | + | ++ | ++ | |
| *Non-parenchymal tissue* | | | | |
| Vascular wall | + | + | + | + |
| Meninges | ++ | ++ | ++ | 0 |
| Choroid plexus | + | + | + | 0 |
| Ventricular ependymocytes | ++ | ++ | ++ | ++ |

The scores of subjective evaluations were ranked as follows: 0 = no signal/cells labelled, + = weak signal, ++ = strong signal, +++ = very strong signal, nd = not determined.
[a]References: Strijbas *et al.* (1991)[18] and Go *et al.* (1994).[10]

### Structures exhibiting the Anx-1 mRNA signal

The areas of the fore- and midbrain which showed a hybridization signal of Anx-1 mRNA are summarized in Table 2. They include the following areas of the telencephalon: layers 2–6 of the cortex, the olfactory bulb, the nuclei of the amygdala, in the hippocampus the pyramidal and molecular layers of the CA1, CA2 and CA3 area and the granular layer of the dentate gyrus, furthermore in the septum and the striatum. Scattered cells were labelled in the hypothalamus, the thalamus and the epithalamus. In the cortex, especially layer 2 of the pyriform and cingulate area showed a high signal intensity (Fig. 2B). Along the fibre bundles of the corpus callosum the oligodendoglia were labelled (Fig. 2A).

No labelling was observed in layer 1 of the cortex and the fimbria of the hippocampus, and notably in the cells constituting the vascular wall, the meninges, the ventricular ependyma, and the choroid plexus (Fig. 2A).

### The Anx-1 mRNA *in situ* hybridization signal in the various groups

The control animals showed labelling of structures shown in Table 2.

The methylprednisolone-treated animals showed Anx-1 mRNA hybridization signals in the same areas as the controls, but far more intense, although the sections had been treated in the same session. Especially the various structures of the hippocampus and the habenular nucleus exhibited a strong increase of signal intenstiy when compared with the controls (Fig. 1). The areas in which Anx-1 mRNA labelling was absent in the controls, such as the cells of the meninges, the ependyma, the vascular wall, and the choroid plexus also lacked Anx-1 hybridization signals in the steroid-treated animals.

Pretreatment with the 21-aminosteroid U74389F gave similar results as pretreatment with methylprednisolone. The only difference was found in the CA3 area of the hippocampus in which Anx-1 mRNA expression was less than

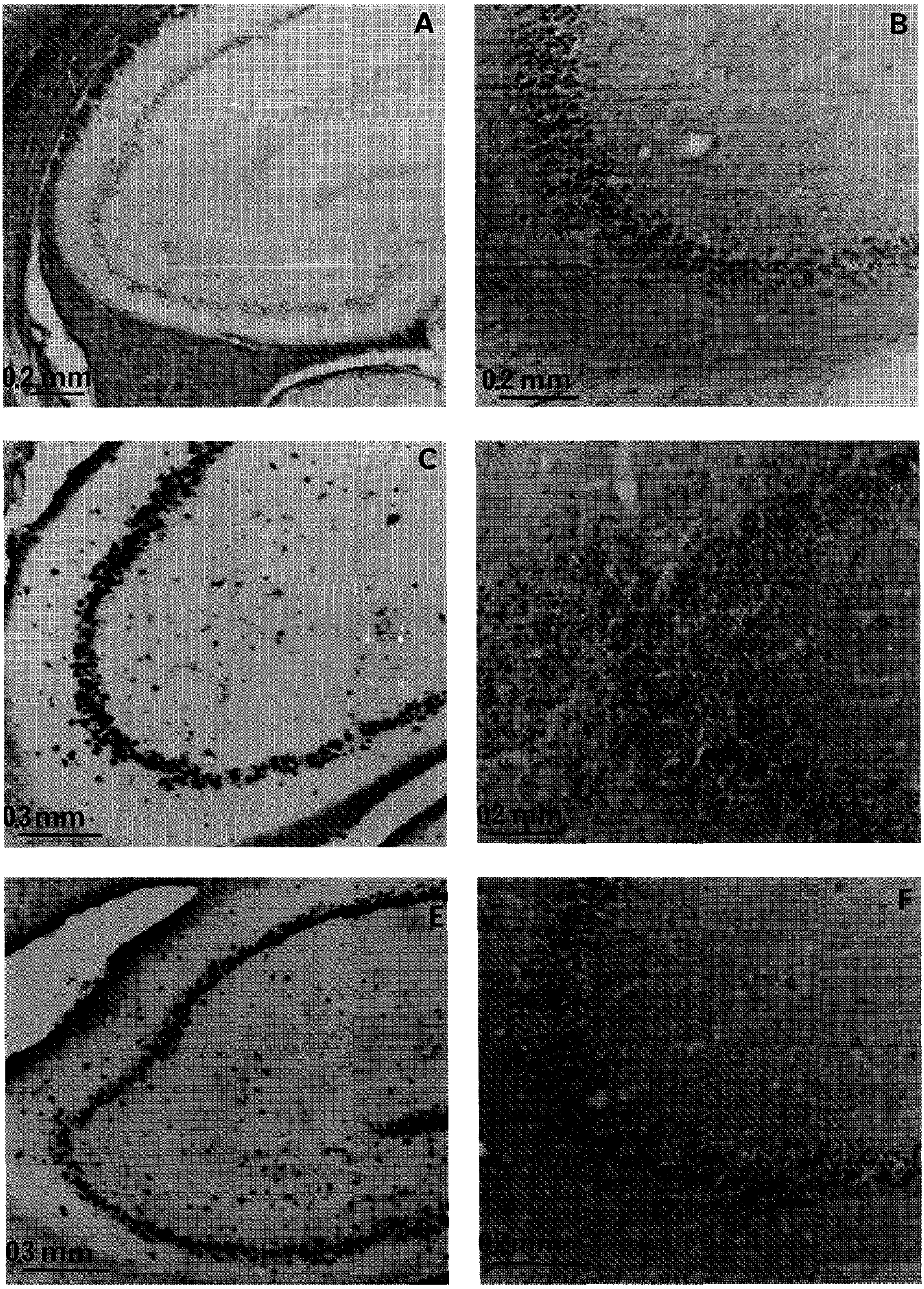

FIG. 1. Photomicrographs demonstrating Anx-1 mRNA by ISH using antisense digoxigenin-labelled cRNA (A, C and E), and immunoreactive Anx-1 (B, D and F) in the hippocampal CA3 area of untreated control (A and B), of methylprednisolone-pretreated (C and D), and of 21-aminosteroid-pretreated rats (E and F).

in the methylprednisolone-treated rats (Fig. 1C and 1E). Again no signal was observed in the cells of the meninges, the ependyma, the vascular wall, and the choroid plexus.

### Immunocytochemistry of Anx-1 protein

In the brains of the control rats, Anx-1 immunoreactivity was observed in layers 2 and 5 of the cerebral cortex, and to a lesser extent in the other cortical layers; furthermore in the basal ganglia, the amygdala, hippocampus, hypothalamus and thalamus (Table 3). The distribution of Anx-1 immunoreactivity was only slightly different from that of Anx-1 mRNA on the ISH: Anx-1 immunostaining of the neuronal cells was most pronounced both in layers 2 and 5, whereas only layer 2 showed the most abundant Anx-1 mRNA expression. In addition, immunoreactive Anx-1 was found in small varicose nerve fibres of neuronal cells, the ventricular ependyma, the choroid plexus, the meningeal cells and sporadically in cells of the vascular wall (Figs 1B, 2C, 2D and 2E). Contrary to the ISH, immunostaining was found in the fibres but not in the oligodendroglia of the corpus callosum.

Methylprednisolone or the 21-aminosteroid pretreatment increased Anx-1 immunoreactivity in other structures, notably the hippocampus, the hypothalamus and thalamus (Fig. 1), but did not change the Anx-1 immunostaining of cerebral cortex, basal ganglia, amydala, septum, meninges, vascular wall, choroid plexus, and ependyma.

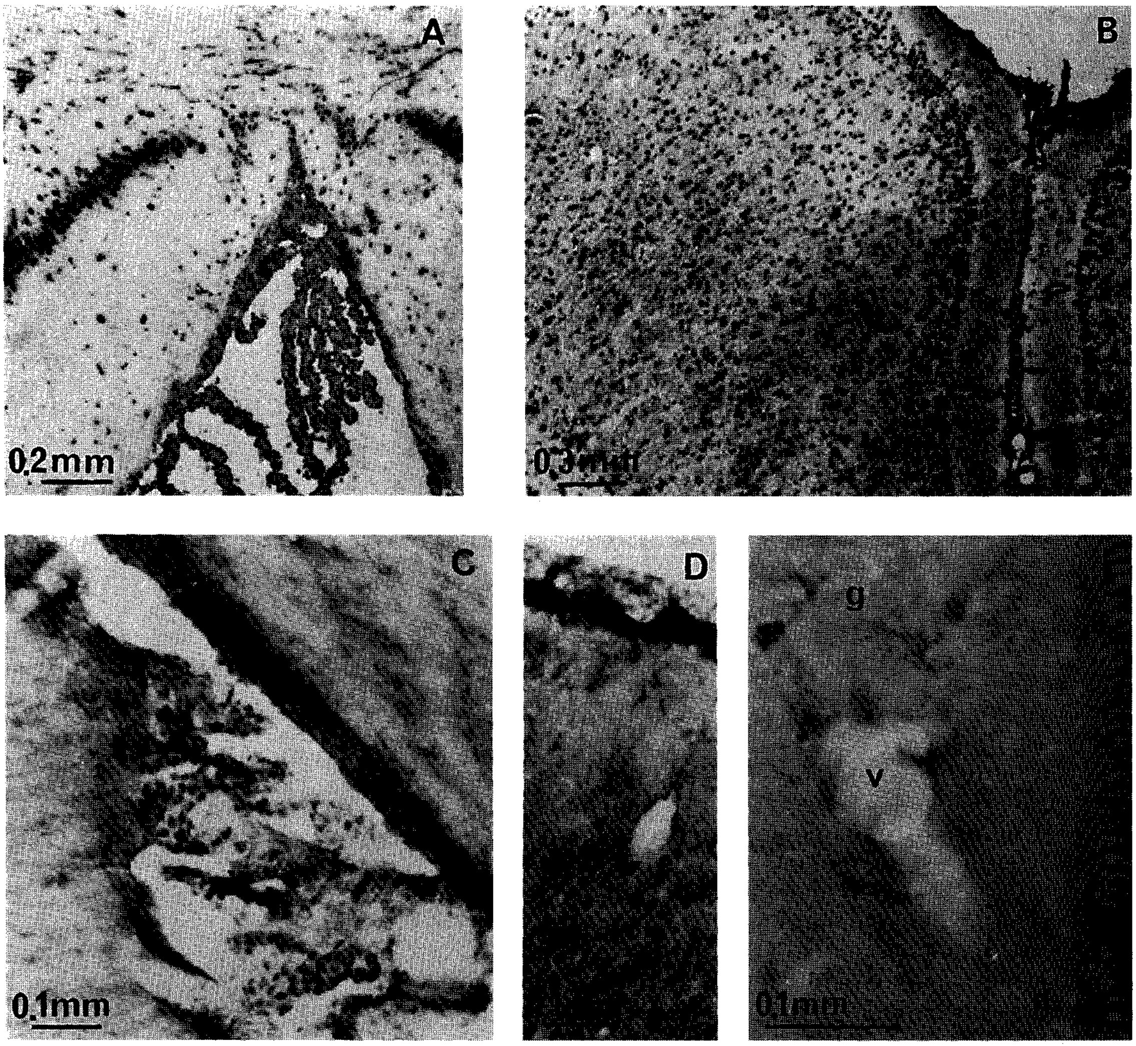

FIG. 2. Photomicrographs of details of ISH and immunoreactive Anx-1 of methylprednisolone-pretreated rats. (A) ISH of the choroid plexus, oligodendroglia in the corpus callosum, and part of the hippocampus. (B) ISH of the cortex, retrosplenial area. Immunohistochemistry of: (C) the choroid plexus, (D) cortex, (E) detail of cortex; glia (g), cells of the vascular wall (v) and neurones (n).

### Discussion

Non-radioactive ISH with digoxigenin-labelled probes as applied in the present study, is a rapid technique with reasonably high spatial resolution. The use of a PCR to incorporate a RNA-polymerase promoter-region in the cDNA constitutes a simple way to produce a template for the RNA-transcription reaction and requires no cloning.[12] Labelling of the probe with digoxigenin-11-UTP also proved a simple and effective procedure. The ISH protocol used in this study has evolved from a number of different protocols.[13–16]

In the present study Anx-1 mRNA has predominantly been shown in neuronal cells within various structures of the brain. Notably, no Anx-1 mRNA has been found in the capillary endothelium and the choroid plexus. Our previous immunocytochemical studies have demonstrated annexins in the choroid plexus of control animals.[10] Also, Anx-1 protein has been demonstrated in choroid plexus and in cellular elements of some other circumventricular organs, such as the median eminence.[17,18] The choroid plexus as well as the other circumventricular organs are notably devoid of a blood–brain barrier. Therefore, the Anx-1 protein observed in the choroid plexus may well be of systemic origin, having entered because of the lack of a blood–brain barrier. This contention seems now to be confirmed by the absence of Anx-1 mRNA in these structures as demonstrated by ISH.

In addition, Anx-1 protein could be demonstrated in the meninges, the ventricular ependyma, and the vascular wall, whereas ISH showed no Anx-1 mRNA transcription. Since these structures are located behind the blood–brain barrier, the protein may have originated by diffusion possibly followed by preferential accumulation from adjacent areas; diffusion of proteins is a well-known and aspecific process in brain tissue.[19] An alternative explanation would be the disparate turn-over rates of mRNA,[20] which may explain subsequent rapid disappearance of the mRNA.

Although in our previous study evidence of Anx-1 immunocytostaining has not been found in the larger part of the brain of control animals, in the present study Anx-1 immunostaining occurred in quite a number of cerebral structures. The differences are most probably attributable to the far greater sensitivity of the free-floating technique, by which in the present study the immunohistochemistry was carried out overnight on 40 μm sections, whereas in the previous study the immunohistochemistry was carried out for a shorter period on tissue sections which had been attached to glass slides. Moreover, the presence of Anx-1 protein in the group of control animals is consistent with the finding of Anx-1 mRNA in the controls, albeit in low, presumably resting levels of transcription. This resting state of Anx-1 mRNA transcription may reflect hormonal influences under physiological conditions, such as the normal glucocorticoid secretion by the adrenals as governed by the hypothalamo-pituitary axis.

The only type of glial cells which could be recognized with reasonable certainty in the present study, without requiring cell-type specific immunocytochemical markers were the oligodendroglia in the white matter. Astrocytes have been shown to express Anx-1 mRNA[21] and to possess glucocorticoid receptors, and may therefore be assumed to be among the labelled cells in the cortex. In the hippocampus, on the other hand, no cells with the morphological features of astrocytes have been found to be labelled.

In the steroid-pretreated animals, there was increased transcription of Anx-1 mRNA in all structures of the brain, as in other tissue.[22,23] Evidence of an increase of Anx-1 immunostaining, however, was only found in some structures, notably the hippocampus, the hypothalamus and thalamus. Another inconsistency was the absence of Anx-1 in the oligodendroglia in spite of ISH evidence of Anx-1 mRNA. It may be speculated that in these cases translation of the Anx-1 protein has not followed the mRNA transcription, presumably impeded by influences yet to be clarified.

Glucocorticoids can cross the blood–brain barrier, and after entering the parenchymal cells bind to the intracellular glucocorticosteroid receptor. The receptor and its mRNA have been demonstrated by immunohistochemistry to occur in nuclei of neuronal and glial cells of the central nervous system with a widespread, but heterogeneous distribution.[24,25] On comparing the distribution of Anx-1 mRNA with that of glucocorticoid receptors (Table 2), a correlation seems to be apparent. The recently introduced 21-aminosteroids have been claimed to exert their action by a direct membrane-protective effect against oxidation by oxygen-derived free radicals,[26] lacking the glucocorticosteroid effect on glucose metabolism; however, our results indicate that they induce expression of annexins as well.

Although in the early 1980s the annexins have been discovered mainly in their role of mediators of glucocorticosteroid anti-inflammatory action by their alleged inhibition of phospholipase $A_2$—with a similar effect on brain oedema being the object of the present study—other properties of the annexins have been revealed in the meantime, including anticoagulant activity (placental Anx-3 and Anx-4), channel activity (Anx-7, Anx-5 and Anx-1), trafficking of vesicles like endocytosis and exocytosis and aggregation of synaptic vesicles (Anx-7, Anx-2, Anx-3 and Anx-4), and involvement in proliferation and differentiation processes (Anx-1 and Anx-2).[27] The recent elucidation of the crystal structures of Anx-5 and Anx-12 has revived the discussion on their role of channels, particularly in view of the hexameric structure of Anx-12.[28,29]

In conclusion the present study has demonstrated the presence of annexin-1 in the brain as a result of local transcription of the Anx-1 mRNA, exhibiting an upregulation following administration of a glucocorticosteroid as well as a 21-aminosteroid. In view of the previously demonstrated anti-inflammatory and anti-oedematous effects of these steroids, this upregulation of annexin expression is presumably involved in mediating these steroid actions on phospholipase $A_2$ activity in the arachidonic acid cascade and eicosanoid formation. Besides, a resting level of annexin expression has been observed which presumably reflects physiological conditions, in which their alleged other functions such as those of channels or in vesicle formation are relevant. It has also been demonstrated by the absence of mRNA transcription, that their occurrence in the choroid plexus can be attributed to a lack of the blood–brain barrier, and that in this respect annexins do not differ from other proteins.

### References

1. Go KG. *Cerebral Pathophysiology. An integral approach with some emphasis on clinical implications*. Amsterdam: Elsevier, 1991: 1–432.
2. Flower RJ. Lipocortin and the mechanism of action of the glucocorticoids. *Br J Pharmacol* 1988; **94**: 987–1915.
3. Parente L, Solito E. Association between glucocorticosteroids and lipocortin 1. *Trends Pharmacol Sci* 1994; **15**: 362.
4. Flower RJ, Rothwell NJ. Lipocortin-1: cellular mechanisms and clinical relevance. *Trends Pharmacol Sci* 1994; **15**: 71–76.
5. Russo-Marie F. Lipocortins: An update (review). *Prostaglandins Leukotrienes and Essential Fatty Acids* 1991; 42: 83–89.
6. Wallner BP, Mattalino RJ, Hesion C, Cate RL, Tizard R, Sinclair LK, Foeller C, Pingchang Chow E, Browning JL, Ramachandran KL, Pepinsky RB. Cloning and expression of human lipocortin, a phospholipase $A_2$ inhibitor with potential anti-inflammatory activity. *Nature* 1986; **320**: 77–81.
7. Becherucci C, Perretti M, Solito E, Galeotti CL, Parente L. Conceivable difference in the anti-inflammatory mechanisms of lipocortins 1 and 5. *Med Inflamm* 1993; **2**: 238–244.
8. Perretti M. Lipocortin-derived peptides. *Biochem Pharmacol* 1994; **47**: 931–938.
9. Moss SE. The annexins. In: Moss SE, ed. *The Annexins*. London: Portland Press, 1992; 1–9.
10. Go KG, Ter Haar JG, De Ley L, Zuiderveen F, Parente L, Solito E, Molenaar WM. The effect of steroid treatment on lipocortin immunoreactivity of rat brain. *Med Inflamm* 1994; **3**: 177–180.
11. Kovacic RT, Tizard R, Cate RL, Frey AZ, Wallner B. Correlation of gene and protein structure of rat and human lipocortin 1. *Biochem* 1991; **30**: 9015–9021.
12. Young ID, Stewart RJ, Ailles L, MacKie A, Gore J. Synthesis of digoxigenin-labeled cRNA probes for nonisotopic in situ hybridization using reverse transcription polymerase chain reaction. *Biotechnic Histochem* 1993; **68**: 153–158.
13. Breitschopf H, Suchanek G, Gould RM, Colman DR, Lassmann H. In situ hybridization with digoxigenin-labeled probes: sensitive and reliable detection method applied to myelinating rat brain. *Acta Neuropathol* 1992; **84**: 581–587.
14. Brouwer N, Van Dyken H, Ruiters MHJ, Van Willigen JD, Ter Horst GJ. Localization of dopamine $D_2$ receptor mRNA with non-radioactive in situ hybridization histochemistry. *Neurosci Lett* 1992; **142**: 223–227.
15. Schaeren-Wiemers N, Gerfin-Moser A. A single protocol to detect transcripts of various types and expression levels in neural tissue and cultured cells: in situ hybridization using digoxigenin-labelled cRNA probes. *Histochemistry* 1993; **100**: 431–440.
16. Wallner BP, Mattalino RJ, Hesion C, Cate RL, Tizard R, Sinclair LK, Foeller C, Pingchang Chow E, Browning JL, Ramachandran KL, Pepinsky RB. Cloning and expression of human lipocortin, a phospholipase $A_2$ inhibitor with potential anti-inflammatory activity. *Nature* 1986; **320**: 77–81.
17. Johnson MD, Kamso-Pratt JM, Whetsell WO, Pepinsky RB. Lipocortin-1 immunoreactivity in the normal human central nervous system and lesions with astrocytosis. *Am J Clin Pathol* 1989; **92**: 424–429.
18. Strijbos PJLM, Tilders FHJ, Carey F, Forder R, Rothwell NJ. Localization of immunoreactive lipocortin-1 in the brain and pituitary gland of the rat. Effects of adrenalectomy, dexamethasone and colchicine treatment. *Brain Res* 1991; **553**: 249–260.
19. Go KG, Houthoff HJ, Hartsuiker J, Van der Molen-Woldendorp D, Zuiderveen F, Teelken AW. Exudation of plasma protein fractions in vasogenic brain edema. In: Inaba Y, Klatzo I, Spatz M, eds. *Brain Edema VI*. New York: Springer, 1985; 76–87.
20. Chen CY, Xu N, Shyu AB. mRNA decay mediated by two distinct AU-rich elements from c-fos and granulocyte-macrophage colony-stimulating factor transcripts: different deadenylation kinetics and uncoupling from translation. *Mol Cell Biol* 1995; **15**: 5777–5788.
21. McLeod JD, Bolton C. Dexamethasone induces an increase in intracellular and membrane-associated lipocortin-1 (annexin-1) in rat brain astrocyte primary cultures. *Cell Mol Neurobiol* 1995; **15**: 193–205.
22. Solito E, Raugei G, Melli M, Parente L. Dexamethasone induces the expression of the mRNA of lipocortin 1 and 2 and the release of lipocortin 1 and 5 in differentiated, but not undifferentiated U-937 cells. *FEBS Lett* 1991; **291**: 238–244.
23. Peers SH, Smilie F, Elderfield AJ, Flower RJ. Glucocorticoid- and non-glucocorticoid induction of lipocortins (annexins) 1 and 2 in rat peritoneal leucocytes in vivo. *Br J Pharmacol* 1993; **108**: 66–72.
24. Cintra A, Zoli M, Rosen L, Agnati LF, Okret S, Wikstrom AC, Gustafsson JA, Fuxe K. Mapping and computer assisted morphometry and microdensitometry of glucocorticoid receptor immunoreactive neurons and glial cells in the rat central nervous system. *Neuroscience* 1994; **62**: 843–897.
25. De Kloet ER, Oitzl MS, Joels M. Functional implications of brain corticosteroid receptor diversity. *Cell Mol Neurobiol* 1993; **13**: 443–455.
26. Hall ED, McCall JM, Yonkers PA, Chase RL, Braughler JM. A nonglucocorticoid analog of methylprednisolone duplicates its high dose pharmacology in models of CNS trauma and neuronal membrane damage. *J Pharmacol Exp Ther* 1987; **242**: 137–142.
27. Raynal P, Pollard HB. Annexins: the problem of assessing the biological role for a gene family of multifunctional calcium- and phospholipid-binding proteins. *Biochim Biophys Acta* 1994; **1197**: 63–93.
28. Huber R, Berendes R, Burger A, Schneider M, Karshikov A, Luecke H, Romisch J, Paques E. Crystal and molecular structure of human annexin V after refinement. Implications for structure, membrane binding and ion channel formation. *J Molec Biol* 1992; **223**: 683–704.
29. Luecke H, Chang BT, Maillard WS, Schlaepfer DD, Haigler HT. Crystal structure of the annexin XII hexamer and implications for bilayer insertion. *Nature* 1995; **378**: 512–515.

ACKNOWLEDGEMENTS. This work was supported by the Jan Kornelis de Cock-Foundation.

**Received 19 August 1996; accepted 6 September 1996**
